# Clinical lipidomics: a new way to diagnose human diseases

**DOI:** 10.1186/s40169-018-0190-9

**Published:** 2018-04-27

**Authors:** Jiapei Lv, Linlin Zhang, Furong Yan, Xiangdong Wang

**Affiliations:** Zhongshan Hospital Institute of Clinical Science, Fudan University Shanghai Medical School, Shanghai Institute of Clinical Bioinformatics, Shanghai, China

**Keywords:** Clinical lipidomics, Disease, Biomarkers, Phenomes, Lipids

## Abstract

Lipidomics is a measurement of a large scale of lipid species to understand roles of their carbon atoms, dual bonds, or isomerism in the lipid molecule. Clinical lipidomics was recently defined “as a new integrative biomedicine to discover the correlation and regulation between a large scale of lipid elements measured and analyzed in liquid biopsies from patients with those patient phenomes and clinical phenotypes”. The first step to translate lipidomics into clinical lipidomics is to settle a number of standard operation procedures and protocols of lipidomics performance and measurement. Clinical lipidomics is the part of clinical trans-omics which was coined as a new emerging scientific discipline where clinical phenomes are integrated with molecular multiomics. We believe it is the time to translate lipid science and lipidomics into clinical application and to understand the importance of clinical lipidomics as one of the most helpful approaches during the design and decision-making of therapeutic strategies for individuals. We emphasize here that clinical lipidomics should be merged with clinical phenomes, e.g. patient signs and symptoms, biomedical analyses, pathology, images, and responses to therapies, although it is difficult to integrate and fuse the information of clinical lipidomics with clinical phenomes. It will be a great achievement if we can draw the networks of lipidomic species fused with networks of genes and proteins to describe the molecular mechanisms of the disease in multi-dimensions.

Lipids are one of the most important elements in cells, tissues, organs, and body, responsible for metabolism, structure, and survival. Lipidomics is a new science of lipids to measure and understand a large scale of lipid species and roles of their carbon atoms, dual bonds, or isomerism in the lipid molecule. A new and extended concept of clinical lipidomics was recently defined “as a new integrative biomedicine to discover the correlation and regulation between a large scale of lipid elements measured and analyzed in liquid biopsies from patients with those patient phenomes and clinical phenotypes” [1]. Different from lipidomics, clinical lipidomics is an new and important merging discipline with a special focus to integrate clinical medicine and lipid science, measure the large scale of lipid species of patient samples, understand disease-specific lipidomic profiles, and fuse with clinical phenomics. Clinical lipidomics has a clear objective to understand mechanisms of systemic metabolisms, identify diagnostic biomarkers and therapeutic targets, and be one of the reliable and repeated approaches to diagnose diseases and monitor therapy. Clinical lipidomics is not just to simply measure a large scale of lipids in patient samples, present a large size of descriptive data, and answer the changes of “higher” or Lower” lipid levels in various diseases. The critical values of clinical lipidomics should be to integrate lipidomic profiles with patient phenomes and clinical phenotypes, to identify disease severity-, duration-, stage-, subtype-, and prognosis-specific lipidomic changes and understand molecular mechanisms of the disease. It would be more valuable if we re-define, re-categorize, and discover diseases, as well as dynamically monitor therapeutic efficacy on basis of clinical lipidomics.

One of the most important steps to translate lipidomics into clinical lipidomics is to settle a number of standard operation procedures and protocols of lipidomics performance and measurement. The amount of lipid species in lipidomics measurement, the number of lipidomics studies on patient samples, and the categories of diseases are increasing. Kim et al. recently measured we all “four major classes of lipids (glycerolipids, phospholipids, sphingolipids, sterols), 17 subclasses of lipids, and 3225 putative individual lipid species in total, as well as a group of dietary lipids” in patients behavioral variant frontotemporal dementia or Alzheimer’s disease, as compared with healthy by liquid chromatography-tandem mass spectrometry [2]. This is one of excellent studies to measure lipidomic profiles in patients with diseases and be compared with other reference diseases which are either in the same category or with high similarity of the target disease. So many excellent clinical studies were performed to measure the lipidomic footprints and characteristics. However, it is hardly to compare “lipidomic profiles” of those studies and have a sharing database of lipidomic results, due to extremely large variation of facilities, methods, protocols, designs, and samplings in lipidomics. There is an urgent need of standard operation protocol for sampling, transporting, storing, and conditioning of individual samples during the clinical practice. Especially, we should have the standard operation and procedure and protocol for sampling tissues from the resection, e.g. time, location, size, operator experience, tissue container, duration, and environmental conditions. We believe that lipidomics is ready for clinical application after the settle of those standards.

It is important to think clinical lipidomics as the part of clinical trans-omics which was coined as a new emerging scientific discipline where clinical phenomes are integrated with molecular multiomics, in order to further understand molecular mechanisms of disease pathogenesis and progression, patient sensitivity to therapy and prognosis, and therapy design and development [3]. Multiomics is defined as a biological analysis approach where more than two omics measurements are performed simultaneously in one cell, organ/tissue, or body, for the identification of disease-specific biomarker and therapeutic targets. Clinical lipidomics is also emphasized to integrate genomics, proteomics, metabolomics, and phenomics. The new concept of clinical lipidomics is not a simple measurement of a large scale of lipid elements in the patients, a simple performance of lipidomics plus clinic information, and a simple correlation between lipid changes and clinical measures. As the part of clinical trans-omics, clinical lipidomics is expected to map lipidomic profiles of patient liquids, cells, and tissues, provide a big data of lipids with clinical phenomes for the database, and explore new mechanisms by which lipids contribute to disease development. One of the major challenges to perform clinical lipidomics is how we can integrate the exact levels and amount of lipid species within lipidomic profiles with clinical descriptive information. We have tried to develop a new digital score evaluation system and translate those clinical descriptive information into digitals in a number of respiratory diseases, although there are still a large number of obstacles to be overcome. We tried to build up the network of genomic or proteomic variants with clinical phenomes by the degree of correlations between [4]. It will be even more challenging to clinical phenomes with lipidomic elements, since each lipid has less specificity and more variations of carbon atoms, dual bonds, and isoform.

More clinical trans-omics are integrated, more values clinical lipidomics will have. We recently measured the profiles of plasma lipidome between health and patients with squamous cell carcinomas, adenocarcinoma, or small cell lung cancer, compared the difference of lipidomic profiles between healthy and patients with lung cancer and between patients with various subtypes of lung cancer, as well as correct lipidomic and genomic profiles of lipid-associated enzymes and proteins by integrating the data of large-scale genome screening [5]. Our preliminary data indicate the clear difference of lipidomic profiles between healthy and patients with lung cancer, e.g. the circulating levels of PS and lysoPS significantly increased, while lysoPE and PE decreased in patients with lung cancer, and the difference of lung cancer-specific and subtype-specific lipidomics. Although this was a preliminary try, the study initially tried to define the difference of lipidomic profiles between cancer subgroups which is a great challenge due to the significance of limited difference between subtypes, the variation of lipid species, or the sensitivity of methodologies. It was also an initial study to link the alterations of lipidomic profiles with the expression of lipid-associated protein genes, although those information was not generated from the same individual, it is hard to address the correlation between enzyme activity and gene expression, and there may be no specificity of the lipid-associated enzymes among lipid species. However, it will be more important to understand mechanisms of systemic metabolisms and identify diagnostic biomarkers and therapeutic targets, if clinical trans-omics can gain enough attention.

Clinical lipidomics as a new science just begins call the attention and action from clinicians and lipid biologists and requires more efforts and strengths than we expect to face and solve potential challenges. Of those, it is questioned whether clinical lipidomics can define disease specificity of dyslipidemia and lipid dysregulation, lipid species have the high specificity of the capacity to monitor one of clinical phenomes, e.g. the severity, stage, duration, response to therapy, and prognosis of patients, or lipid species as biomarkers can be monitored dynamically. Due to the complexity and comprehensive understanding of lipid science, the application of clinical lipidomics will be also questioned and criticized by lipid biologists, since the choice and decision of lipidomic measurements may be not optimal and perfect according to the opinions of biologists. The method of lipidomic measurement for clinical performance should be more stable, repeatable, simple, and sensitive, as other measures in clinic. It will be other challenge to educate and update clinicians about the importance of clinical lipidomics in understanding of disease diagnosis and pathophysiology and patients about the meaning of lipidomic profiles.

In conclusion, it is the time to translate lipid science and lipidomics into clinical application and to understand the importance of clinical lipidomics as one of the most helpful approaches during the design and decision-making of therapeutic strategies for individuals. We emphasize here that clinical lipidomics should/must be merged with clinical phenomes, e.g. patient signs and symptoms, biomedical analyses, pathology, images, and responses to therapies, although it is difficult to integrate and fuse the information of clinical lipidomics with clinical phenomes. It will be a great achievement if we can draw the networks of lipidomic species fused with networks of genes and proteins to describe the molecular mechanisms of the disease in multi-dimensions.
